# Changes in mean body size in an expanding population of a threatened species

**DOI:** 10.1098/rspb.2022.0696

**Published:** 2022-06-08

**Authors:** Graeme C. Hays, Albert Taxonera, Berta Renom, Kirsten Fairweather, Anice Lopes, Jacquie Cozens, Jacques-Olivier Laloë

**Affiliations:** ^1^ School of Life and Environmental Sciences, Deakin University, Geelong, Victoria, Australia; ^2^ Project Biodiversity, Santa Maria, Sal, Cape Verde; ^3^ SOS Tartarugas, Santa Maria, Sal, Cape Verde

**Keywords:** harvesting, trophy-hunting, population recovery, conservation success

## Abstract

With some taxa, a reduction in the mean size of individuals may reflect over-harvesting and/or trophy hunting. However, we show that in sea turtles, a reduction in the mean size of breeding individuals may be part of the good news story of an expanding population. We describe a 70-fold increase in annual nest numbers on the island of Sal (Cape Verde, North Atlantic) between 2008 and 2020 (from 506 to 35 507 nests), making this now one of the largest loggerhead (*Caretta caretta*) nesting aggregations in the world. We use 20 128 measurements of the size of nesting turtles to show that their mean annual size has decreased by about 2.4 cm, from 83.2 to 80.8 cm. This decrease in the mean size of nesting turtles was not caused by the removal of larger turtles, for example by selective harvesting. Rather we develop a theoretical model to show than this decrease in mean size can be explained by an influx of first-time nesters, combined with a decrease in the size of those first-time nesters over time. A reduction in mean size of nesting turtles has been reported across the Atlantic, Pacific and Indian Oceans, and may be a common feature of population recoveries in sea turtles.

## 1. Introduction

Across the world's oceans there are many well-recognized threats to biodiversity including climate change, overharvesting of resources, habitat loss and invasive species that have led to declines in abundance across multiple taxa, including extinctions [1]. Accompanying changes in abundance, there may sometimes be changes in the mean size of individuals. For example, major reductions in the size at maturity have occurred for some exploited fish species [2,3] as well as with trophy-hunting such as for the horns or tusks of some mammals [4,5]. Set against that backdrop, there is cause for measured optimism with ongoing conservation efforts for some taxa and habitats proving very successful [6]. For example, for 124 well-assessed marine mammal populations, 47% have shown significant increases over recent decades and only 13% are decreasing [6]. Some populations of humpback whales, northern elephant seals and southern sea otters are among the most notable increases in abundance [6,7]. Similarly, many populations of sea turtles have shown recent increases in abundance, with significant upwards trends reported at 95 individual nesting sites versus 35 significant decreases [8]. For sea turtles, upward trends in abundance have been recorded in a range of species and across the globe. There are likely to be a range of ecosystem and demographic consequences of such population recoveries [6] with the resulting trophic cascades sometimes reshaping communities [9,10]. For example, increasing numbers of green turtles have been associated with overgrazing of seagrass beds [10]. However, overall the demographic changes occurring as part of species recoveries are poorly understood.

Here we use long-term observations at one of the world's largest sea turtle rookeries to identify how both the mean size of individuals and the population size have changed. At a number of sea turtle nesting areas around the world, reductions in the mean size of individual have been reported as the population size has increased (e.g. [11]). These observations are enigmatic, since declining mean size in other taxa is often linked to overharvesting and population declines [2,4], rather than population increases. One hypothesis to explain the reduction in mean turtle nester size over time is that an increased number of first-time nesters, which are often smaller than experienced nesters, are driving both the increase in nesting numbers as well as the reduction in mean size [11]. Here we test this hypothesis by developing a model that links population increases with body size via demographic shifts. In this way, we assess whether a reduction in mean size may be a common feature across the world at the many sites where nesting numbers are increasing.

## 2. Material and methods

### (a) Study site and beach monitoring

**Figure 1.**
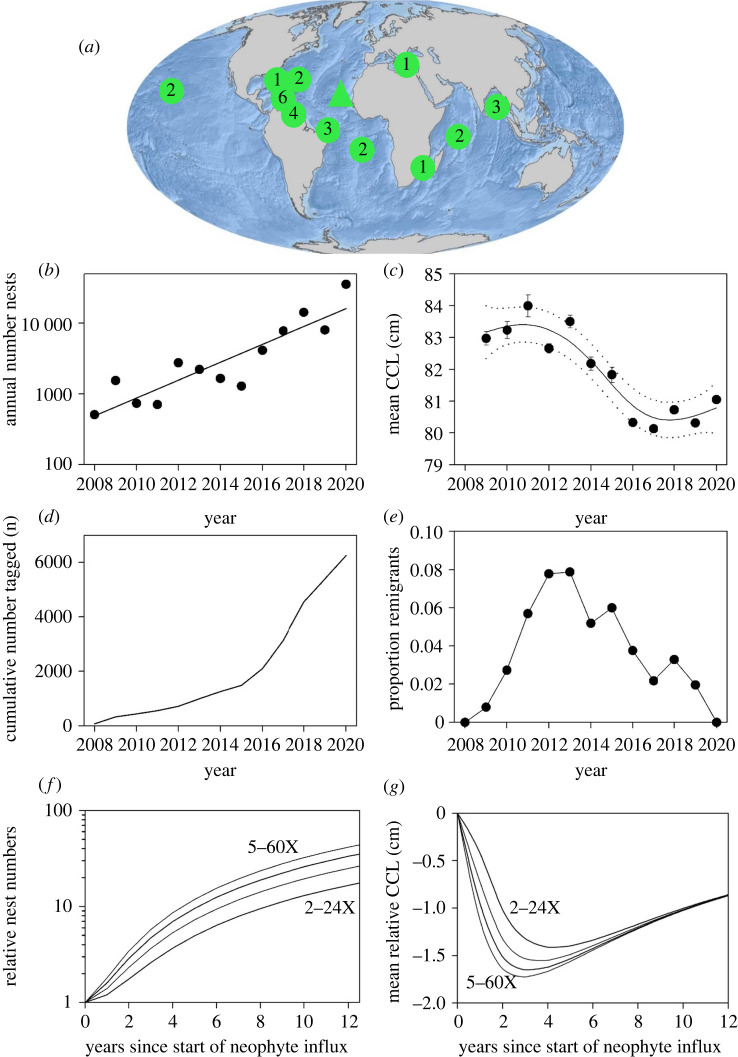
(*a*) The location of Cape Verde (triangle) and regional management units (RMUs) around the world where the number of nests is increasing (adapted from Mazaris *et al*. [8]). 1 = loggerhead turtle (*Caretta caretta*), 2 = green turtle (*Chelonia mydas*), 3 = olive ridley (*Lepidochelys olivacea*), 4 = hawksbill (*Eretmochelys imbricata*), 5 = Kemp's ridley (*L. kempii*), 6 = leatherback (*Dermochelys coriacea*). (*b*) The annual number of nests deposited on Sal increased markedly between 2008 and 2020. The model line of log number of nests versus year explained 81% of the variance (*F*_1,11_ = 44.3, *r^2^* = 0.81, *p* < 0.01). (*c*) There was a general decrease in mean annual CCL between 2009 and 2020 (approximate significance of the GAM smooth: *p* < 0.01, *F*_3,6_ = 16.1). Annual means ± 1 SE are shown. The solid line is the cubic smoothing spline fitted using a GAM and the dashed lines represent the 95% confidence envelopes. See electronic supplementary material, figure S3 for histograms of showing the shift in size from the start to the end of the time series. (*d*) The cumulative number of turtles flipper tagged versus year. (*e*) The proportion of turtles encountered with flipper tags from a previous year (i.e. remigrants) as a proportion of the number of turtles seen with flipper tags that year (remigrants plus flipper tagged neophytes). (*f*) The modelled increase in nesting numbers assuming annual survival rate of 0.8 and a progressive increase in the number of neophytes entering the nesting population, from 2–24×, 3–36×, 4–48× and 5–60× compared to the initial value. So after 12 years the number of neophytes entering the nesting population had increased 24×, 36×, 48× and 60× in each model run, compared to the starting value. (*g*) Under the scenarios in panel *d*, the decrease in mean nester size.

The Cape Verde archipelago (figure 1*a*) is a group of ten islands that are roughly 600 km off the coast of West Africa (14.8–17.2° N and 22.7–25.4° W). Loggerhead turtles (*Caretta caretta*) nest across the archipelago, with the species listed globally as ‘vulnerable’ [12]. Data were collected from the island of Sal in the northeast of the archipelago, during the turtle nesting season (June–October). Regular beach surveys were conducted nightly to record turtle nesting activity. The complete methodologies are described in detail in [13]. In short, we conducted night surveys (20.00 to 06.00) on the island's main nesting beaches during the nesting season. Observers walked along the high-water mark and recorded turtles and turtle tracks encountered. Observers were trained to identify presence/absence of nest when a turtle track was found. If a turtle was encountered, the turtle was observed discreetly to record whether it nested. Morning surveys were systematically conducted after night surveys to confirm no activities were unaccounted for.

In addition to nightly beach surveys of the main nesting beaches, all the island's beaches were surveyed fortnightly. Over the course of one day, all turtle tracks found on the island were inspected and the type of activity (nest versus no nest) was recorded. A correction factor was applied to account for the expansion of monitoring over the 13-year period with more beaches being surveyed in the recent years (see [13]). In 2019 and 2020, due to unprecedented numbers of nesting activities, the total number of nests produced across two weeks could not be recorded accurately. Instead, the number of ‘fresh’ activities (i.e. activities from the previous night) were counted and then the nightly number of nests was interpolated between these fortnightly records.

### (b) Biometric measurements

When turtles were encountered during night surveys, we measured their curved carapace length (CCL) and their curved carapace width (CCW) using a flexible tape measurer. To avoid disturbing the nesting process, turtles were measured only if they were nesting and only after oviposition started. CCL was measured from the back of the turtle's neck where the skin meets the nuchal scute to the posterior-most tip of the carapace. CCW was measured at the widest part of the carapace. CCL and CCW were each measured three times and a mean was then calculated for each. In 2016 the methods for measuring CCL changed to conform to how turtles were being measured on the other islands of the Cape Verdean archipelago. After 2016, CCL was measured from the nuchal scute to the notch where the two most posterior marginal scutes meet. In 2016, both ‘CCLtip’ and ‘CCLnotch’ measurements were taken. We used a simple linear model to estimate CCLnotch from CCLtip for 2017, 2018, 2019 and 2020 (electronic supplementary material, figure S1; *p* < 0.01, *r*^2^ = 0.98, *n* = 786 pairs of measurements). In this manner, we present CCLtip (hereafter referred as CCL) measurements between 2008 and 2020. In addition, when possible, the number of eggs deposited in the clutch was recorded.

### (c) Tagging and growth rates

Nesting turtles were tagged externally with Inconel flipper tags from 2008 to 2018. Two tags were applied to each turtle, one in the trailing edge of each front flipper. The tags were placed between the second and third scales closest to the body of the turtle. Turtles were systematically checked for tags after nesting. When tags were resighted, their unique identifying numbers were recorded. If a turtle was found with only one tag, a new tag was applied to replace the missing tag. New tags were applied between the third and fourth scale closest to the body of the turtle on the same flipper from which the tag was missing. In addition, we checked flippers for scars that might be indicative of tag losses on turtles that did not have any tags. Additionally to flipper tagging, we applied PIT tags to turtles in 2008 and between 2013 and 2020.

Using CCL and CCW measurements in combination with tagging records we estimated growth rates for adult nesting females. We calculated growth rate in centimetres per year as follow: Growth rate = (size of turtle when last seen − size of turtle when first seen)/(time between the first and last sighting of the turtle). We only calculated growth rates for turtles that had a minimum period of 1 year between their first and last sighting. Across all years, a large number of turtles were tagged (*n* = 6252) but this number was still relatively small compared to the total number of nests (*n* = 80 983). So while we could track the growth of the marked turtles over time, we could not assess differences in the size of first-time nesters (neophytes) versus those turtles returning for their second and subsequent nesting seasons (remigrants).

### (d) Long-term trend analysis

We analysed long-term trends in mean CCL and mean CCW using generalized additive models (GAMs). We used the cross-validation method to estimate smoothing parameters. We analysed annual number of nests using generalized additive models (GAMs) and used the cross-validation method to estimate smoothing parameters. We used a negative binomial error distribution for the analysis of number of nests. These analyses were done in R v. 4.0.3 [14] and GAMs were implemented using the mgcv library.

To assess changes in the abundance of turtles of different size classes, we used the proportions of turtles measured in different size classes each year multiplied by the total number of nests each year.

### (e) Sea surface temperature on foraging grounds

A dichotomy in foraging habitats has been shown through satellite tagging of nesting loggerhead turtles nesting on the island of Boa Vista, Cape Verde, an island 40 km south of Sal, with smaller turtles (CCL < 90 cm) foraging in the open ocean between Cape Verde and West Africa and larger turtles (CCL > 90 cm) foraging on the coast of West Africa [15]. Based on satellite tracking results [15], key oceanic and coastal foraging areas were defined by areas borders by 15–17° N, 17–19° E and 7–9° N, 13–15° E, respectively. For each area, sea surface temperature (SST) data were obtained from the International Comprehensive Ocean-Atmosphere Data Set (ICOADS) through the National Center for Atmospheric Research (NCAR) (https://rda.ucar.edu/datasets/ds548.0/)

### (f) Modelling changes in body size

Within a quantitative framework, we explored how an influx of neophytes alone might change the mean size of the nesting turtles, i.e. the mean size combined for both neophytes remigrants. We first considered a ‘start of time-series' population where the same number of neophytes entered the population each year for many decades. We parametrized the model using annual survival of 0.7, 0.8 and 0.9, typical values reported around the world [16]. Turtles continue to grow after reaching sexual maturity. We parametrized annual growth rates based on our measurements of mean annual growth rates, combined with the age-dependent growth rates reported by [17], who reported the annual growth rate for nesting loggerheads declined linearly as they aged, in the first year of nesting being about 1.4× the mean annual growth rate for the nesting population, decreasing down to zero about 14 years after first nesting. We recorded a mean growth rate of nesting turtles of 0.64 cm y^−1^ (see Results) and therefore parametrized our model by assuming that in the 14 years after first nesting, the growth rate of turtles declined from 0.9 cm y^−1^ to zero. We increased the annual number of neophytes progressively over 12 years, to assess how an influx of neophytes might change the annual number of nests as well as the mean size. So, for example, using a modelled annual survival of 0.8, then of the neophytes in the population in year 0, the proportion surviving in years 1, 2, 3 etc. would be 0.8, 0.8^2^ = 0.64, 0.8^3^ = 0.512 and so on. So, for example, if there were 100 neophytes in the population in year 0, the number surviving in years 1, 2 and 3 would be 80, 64, 51.2 and so on. Compared to the initial ‘start of time-series’ value, the number of neophytes was increased in successive years by 2×, 4×, 6× up to 24× after 12 years. So, for example, if there were 100 neophytes in the population in year 0, then the number entering in years 1, 2, 3 etc. would be 200, 400, 600 up to 2400 in year 12. Similarly, we assumed that the number of neophytes increased each year from 3–36×, 4–48× and 5–60× over 12 years. See electronic supplementary material for an example spreadsheet of the initial model set up and code to generate the time-series of mean annual size. These calculations were run in Minitab v. 8.2 extended.

## 3. Results

The annual number of nests on Sal has increased rapidly from 506 nests in 2008 to 35 507 nests in 2020, a 70-fold increase. This increase is well described by a model fit of log number of nests versus year (figure 1*b*). Between 2009 and 2020, the carapace length of turtles was measured on 20 128 occasions (electronic supplementary material, table S1). Accompanying the increase in nest numbers, the mean annual size of nesting turtles decreased significantly, with the fitted model describing the decrease in CCL from 83.2 cm in 2009 to 80.8 cm in 2020, a 2.4 cm decrease over 11 years (figure 1*c*). Similarly mean annual CCW decreased across years (electronic supplementary material, figure S2). The number of eggs laid was recorded for 6486 clutches (electronic supplementary material, table S2). The mean clutch size was 79.5 eggs (s.d. = 15.5) and tended to increase with CCL, although the relationship was weak (*r*^2^ = 0.11, *F*_1,6484_ = 793, *p* < 0.001). The predicted mean size of clutches for turtles of 83.2 cm and 80.8 cm were 82.7 and 79.7 eggs respectively, a 3.6% decrease.

The cumulative number of turtles that had been flipper tagged as the time series progressed increased from 76 turtles at the end of 2008 up to 6252 turtles by the end of 2020 (figure 1*d*). If returning turtles had mostly nested in previous years (i.e. were remigrants), then the proportion of turtles observed with tags as a function of annual nest numbers would be expected to increase through the time series. However, this was not the case. The proportion of observed turtles with tags from a previous year initially increased until 2011, reflecting the turtles tagged at the start of the time series returning to nest. Then after 2011 the proportion of tagged remigrants then progressively decreased to close to zero (figure 1*e*).

The mean interval between repeat CCL measurements of the same individuals was 2.8 years (*n* = 93 repeat measurements, range 1.1–7.9 years, s.d. = 1.2 years). The mean growth rate of nesting turtles was 0.64 cm y^−1^ (*n* = 93, s.d. = 6.54 cm y^−1^) equivalent to 0.78% y^−1^. We therefore estimated that in the 14 years after first nesting, the growth rate of turtles declined from 0.9 cm y^−1^ to zero. Under all the modelled scenarios for the influx of neophytes, the annual number of nests increased markedly and the mean size of turtles initially decreased before increasing (figure 1*f*,*g*; electronic supplementary, figures S3, S4). For example, assuming an annual survival of 0.8 and a progressive 4–48× increase in the number of neophytes, the size of the nesting population increased 33-fold over 12 years and the annual mean size decreased maximally by 1.65 cm (figure 1*f*,*g*). When the influx of neophytes was greater, the modelled increase in the size of the nesting population tended to be greater, as expected, and the initial decline in annual mean nester size tended to be larger. However, the same general patterns in population growth and changes in mean nester size were evident across all modelled scenarios.

Our models predict that as neophytes enter the population, nest numbers increase and mean size goes down. But then as those neophytes continue to grow and return to nest in subsequent years, so nest numbers continue to increase but now those neophytes are returning as larger individuals since they have grown a little since they first nested, and so the mean size first stabilizes and may then even increase over time (figure 1*g*; electronic supplementary material, figure S4, S5). This model prediction has some support in our empirical data with mean size appeared to stabilize after 2016 (figure 1*c*). When the modelled increase in population size over 12 years was between 25-fold and 40-fold, the modelled decrease in annual mean nester size reached maximum values of between 1.11 and 2.49 cm.

**Figure 2.**
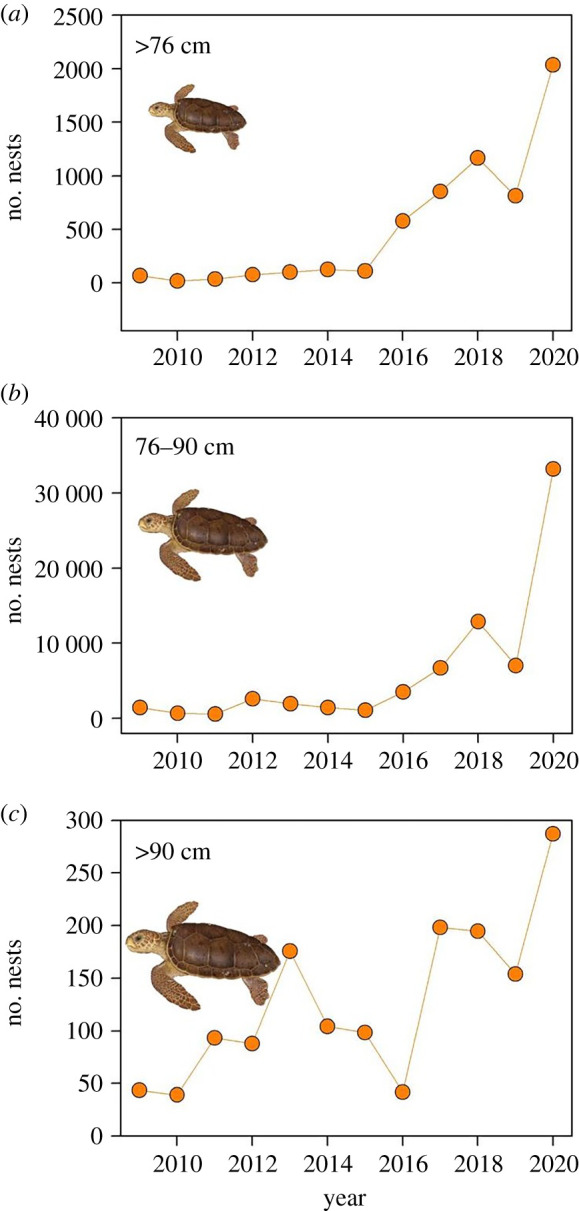
The number of nests each year for turtles in three size classes. (*a*) less than 76 cm CCL, (*b*) 76–90 cm CCL and (*c*) greater than 90 cm CCL. There was a marked increase in numbers in all size categories, although the increase was least marked for the largest size category. Note the different *y*-axis scales. For turtles in size classes less than 76 cm CCL, 76–90 cm CCL and greater than 90 cm CCL, the number of nests increased by 31-fold, 23-fold and 7-fold respectively. (Online version in colour.)

The number of nests increased in all size classes as the time-series progressed (figure 2). For example, between 2009 and 2020 the number of nests for turtles in size classes less than 76 cm, 76–90 cm and greater than 90 cm, increased by 31-fold, 23-fold and 7-fold respectively.

**Figure 3.**
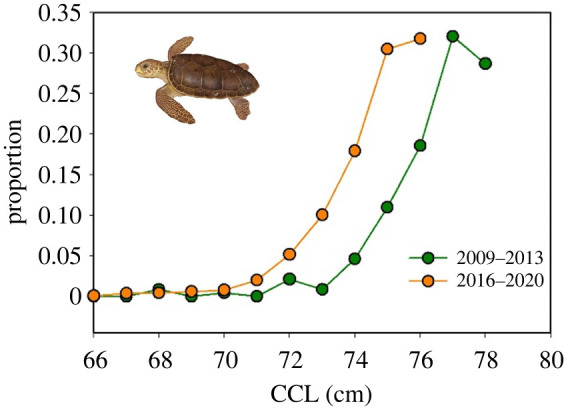
The shift in the size distribution of the smallest 10% of turtles between the first and last 5-years of the time series (green circles = 2009–2013, orange circles = 2016–2020). (Online version in colour.)

As well as a decrease in the mean size of turtles through the time-series, there was also a decrease in the size of the smallest turtles in the nesting population (figure 3). For example, between the first and second halves of the time-series (2009–2013 versus 2016–2020), the mean size of the smallest 10% of turtles decreased significantly by 1.7 cm from 76.3 cm (s.d. = 2.0 cm, *n* = 237) to 74.6 cm (s.d. = 1.7 cm, *n* = 1661) (*t*_288_ = 12.3, *p* < 0.0001) (figure 3).

**Figure 4.**
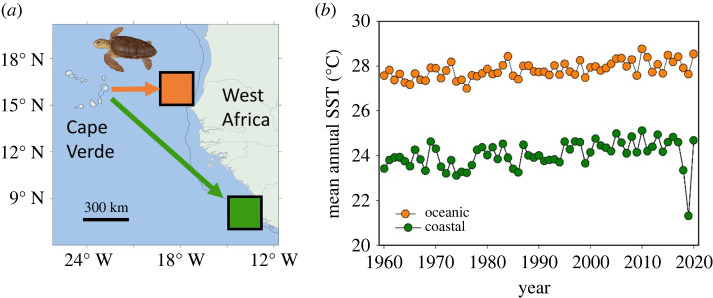
Long-term trends in the mean annual SST in the coastal foraging sites inhabited by turtles greater than 90 cm CCL (green symbols) and the oceanic foraging sites inhabited by turtles less than 90 cm CCL (orange symbols). In both time-series there was a significant increase in temperature over time (*F*_1,59_ > 6.8, *p* ≤ 0.01) with temperatures increasing about 0.1°C per decade. (Online version in colour.)

Sea surface temperatures were warmer in the oceanic versus coastal foraging areas and in both areas there was a long-term increases in SST, with increases in both areas of about 0.1°C per decade between 1960 and 2020 (figure 4). However, the change in mean turtle size across years was not related to either the sea surface temperature in the oceanic foraging habitat (*F*_1,10_ = 0.04, *p* = 0.842) or neritic foraging habitat (*F*_1,10_ = 1.74, *p* = 0.216) used by turtles nesting in Cape Verde.

## 4. Discussion

Contrasting with well-known examples such as overfishing and trophy-hunting, where a reduction in the mean size of individuals often reflects overharvesting [2,4], the decrease in the mean size of nesting turtles we recorded was not caused by the removal of larger size classes of turtles, since the number of nests increased over time for all size classes. Rather the reverse appears to be the case, with the size reduction of adults linked, to some extent, to the positive conservation outcome of a rapidly expanding population. This increasing nesting abundance in Cape Verde has been attributed, at least in part, to the onset of protection on nesting beaches so that fewer nesting turtles were harvested [13]. This protection of adults will not only increase the annual survival rate of adults but will also increase the number of eggs being laid each year both of which may lead to long-term increases in population size. Although estimates are uncertain, loggerhead turtles in the Atlantic are thought to reach maturity at around 20–40 years old [18]. As such, the increase in nesting numbers might indicate increased hatchling survival many years before the observed increase in nesting numbers.

Our calculations are informative as they show how an influx of neophytes that causes the annual number of nests to increase will likely always be accompanied by a decrease in mean nester size if neophytes are generally smaller than remigrants. This is a reasonable assumption since we show turtles continue to grow after reaching maturity. It might be argued that the individual variability within a population in the size of turtles at which they attain maturity [18] might mask a difference in size between neophytes and remigrants. Nevertheless, this scenario appears to often not be the case as differences in the size of neophytes versus remigrants have been demonstrated many times both for loggerhead turtles and other species [19–21]. For example, in the eastern Mediterranean the carapace length of neophyte versus remigrant loggerhead turtles were 87.7 cm versus 92.0 cm respectively [20]. Our calculations suggest that some reduction in mean size will generally occur with a long-term increase in nesting numbers. There are limited sites to test this prediction, since while many marked long-term increases in turtle nesting numbers have been described [8], the annual mean size of individuals is often not reported. Nevertheless, when both nesting numbers and mean size have been reported, a decrease in mean size accompanied an increase in the number of nests for nesting sites in the Atlantic (green turtles at Ascension Island [11], loggerhead and green turtles in Florida [22], the Pacific (green turtles in Hawaii [23]) and the Indian Ocean (loggerhead turtles in South Africa [21]).

It should be noted that we have only constructed very simple models whose outcomes only approximate the observations. Further work could elaborate these models. In particular, relatively few turtles were tagged in proportion to the number of nests, largely due to the huge volume of nesting turtles and hence the logistic challenge of intercepting a high proportion of turtles while they were ashore. In other regions where populations are expanding and the mean turtle size decreasing [11,21,22], it may be possible to tag a greater proportion of nesting turtles and so, for example, more clearly identify the relative numbers of neophytes versus remigrants as well as any possible changes in breeding intervals.

It is noteworthy one of the model predictions is that as an influx of neophytes grow and age, then after several years the mean nester size is predicted to start to increase back toward starting values. We saw support for this model prediction in our data, where mean size did not continue to decrease at the end of the time series. Further the predicted pattern of mean size returning to starting levels has been recorded with one of the longest time-series available (30+ years) for green turtles nesting in Hawaii [23], which provides further support for the general applicability of our predictions. These considerations suggest that turtle shrinkage may be a universal feature of expanding populations. In theory, the increase in nesting numbers we observed could have been driven by an influx of turtles formerly nesting elsewhere whose beaches have been lost, e.g. due to development or erosion. However, there is no evidence to support this idea with no reported decreases in nesting numbers elsewhere in the Cape Verde archipelago [13]. The interval between breeding seasons in loggerheads is thought to generally be two or more years [16]. A shortening of this remigration interval might account for some of the increase in nesting numbers, although there is no evidence for such a shortening.

In addition to an influx of neophytes causing nest numbers to increase and mean nester size to decrease, the shift in the size distribution of turtles also suggests that neophytes have become smaller over time. The reasons for a decrease in neophyte size are unknown, but in other taxa, such as fish, there is evidence that climate change may drive long-term changes in the size at maturity [24]. In fish and other ectotherms, the size at maturity tends to be smaller at warmer temperatures [25,26]. Across terrestrial turtles, body size has been reported to be linked to temperature, but no such relationship was found in aquatic freshwater turtles [27]. For loggerhead turtles the observed change in body size was not linked to temperature on the foraging grounds, which suggests that other factors, aside from temperature, primarily drive the change in mean body size that we observed. Other ecosystem changes, such as food availability, may contribute to the long-term changes in mean nester size and may explain why changes in turtle nester size have occurred at some nesting sites in the absence of increases in abundance [28]. We can make different predictions about how mean nester size might change in the future depending on the relative importance of simply an influx of neophytes versus a shift in climate. If there is simply an influx of neophytes our calculations suggest that after an initial decline the mean nester size will return towards original values as has been seen at some sites [23]. In contrast, if climate change is the principal driver of decreases in mean size, then as climate change accelerates we might expect further decreases in mean nester size.

Our results add to the growing reports of marked increases in nesting numbers at sites across the globe. Protection of nesting turtles and their eggs are likely to have helped drive these population increases [8]. Little is known about the foraging ecology and threats that turtles nesting in Cape Verde face in their foraging areas in oceanic north Atlantic and the coast of West Africa, although bycatch in commercial fisheries have been flagged as a general threat for loggerhead turtles [16,29]. As well as reduced harvesting while nesting, reduced mortality in their foraging areas may be a further reason for the increase in nest numbers, particularly in oceanic areas since the increase in numbers of smaller turtles has been the greatest. Previously the largest loggerhead turtle rookery was thought to be in Oman (Masirah Island), with the most recent estimates of 55 000 nests per year using survey data up to 2016 [30]. So with a total of 35 000 nests on in 2020, Sal now probably hosts one of the largest loggerhead turtle aggregations in the world with considerably more turtles across the archipelago as a whole [31].

While we predict a decrease in mean size might be widely occurring around the world associated with increases in nest numbers, the broader demographic and ecosystem consequences of a decline in mean nester size are not fully known. Since smaller individuals tend to lay fewer eggs per clutch (e.g. our study, [32,33]), rates of increase in total egg production will be slightly less than increases in nesting numbers, although our results suggest this difference is likely to be only a few percent. Furthermore, it is likely that tens of thousands of nests per year on Sal, and in Cape Verde more broadly, translate to many hundreds of thousands of turtles, i.e. adults and juveniles, foraging in the open ocean, which may be altering pelagic food webs.

For some taxa, including fish and some freshwater turtles, the dispersal ability correlates positively with body size [34,35] and survival may be linked to dispersal ability [36]. So for some taxa, a reduction in size may equate with a reduction in fitness. However, for sea turtles their dispersal as hatchlings is largely driven by ocean currents and so turtles can disperse widely across ocean basins regardless of their size [37]. Sea turtles are then thought to use, as adults, foraging areas they encountered as drifting hatchlings [37]. So the changes in mean body size that we reported for loggerhead turtles are unlikely to impact their survival.

In conclusion, our results show a remarkable increase in nest numbers on the island of Sal, Cape Verde, and accompanying the influx of first time-nesters, which likely explains this population expansion, there has been a marked decrease in the mean size of nesting turtles. This shrinkage of marine turtles has also been observed at sites across the Atlantic, Pacific and Indian Oceans and may often reflect the good news story of widespread population recoveries across the world [8].

## Data Availability

The dataset supporting this study is stored in the electronic supplementary material [38] and the main text.
